# Dual Burden of Underweight and Overweight among Women in Bangladesh: Patterns, Prevalence, and Sociodemographic Correlates

**Published:** 2015-03

**Authors:** S.M. Mostafa Kamal, Che Hashim Hassan, Gazi Mahabubul Alam

**Affiliations:** ^1^Department of Mathematics, Islamic University, Kushtia 7003, Bangladesh and Visiting Research Fellow, Unit for the Enhancement of Academic Performance, University of Malaya, 50603 Kuala Lumpur, Malaysia; ^2^Unit for the Enhancement of Academic Performance, University of Malaya, 50603 Kuala Lumpur, Malaysia

**Keywords:** Dual burden, Malnutrition, Overweight, Underweight, Bangladesh

## Abstract

The discourse of dual burden caused through underweight and overweight is well-documented globally but this issue and its connection with women's health in Bangladesh is yet to be explored widely. To enrich the current debate, this study, in the context of Bangladesh, examines the patterns, prevalence, and socioeconomic factors influencing the ever-married women of being underweight and overweight over normal weight. Data used in this study have been extracted from the most recent 2011 Bangladesh Demographic and Health Survey. To achieve results connected with the research objectives, both bivariate and multivariate statistical analyses have been employed. In bivariate analysis, we used seven categories of BMI cutoff points for Asian countries as prescribed by World Health Organization (WHO). Multinomial logistic regression model was constructed to investigate the net effect of socioeconomic factors on underweight, pre-overweight, and overweight over normal weight. The results confirm the co-existence of underweight and overweight among women as we found the prevalence of underweight, normal weight, pre-overweight, overweight, and obesity to be 24.1%, 46.7%, 12.8%, 13.5%, and 2.9% respectively. Compared to the richest, the women from the poorest households were significantly (p<0.001) most likely to be underweight (OR=2.75, 95% CI 2.27-3.35) and least likely to be overweight (OR=0.15, 95% CI 0.12-0.19) over normal weight. The urban women, compared to their rural counterparts, were significantly (p<0.001) less likely to be underweight (OR=0.80, 95% CI 0.71-0.91) and more likely to be overweight (OR=1.33, 95% CI 1.18-1.51) than normal weight. The other socioeconomic grades that were most marked to be underweight and overweight are age, women's education, marital status, age at first childbirth, parity, number of children aged ≤5 years at the household, and food security. The findings confirm the dual burden of both under- and overweight. Systematic and regular monitoring and surveillance of the social trajectory of nutritional status of women and men in Bangladesh is crucial to develop apposite strategy that addresses the persistent and chronic problem of underweight and the emerging problem of overweight. The dual existence of both types of malnutrition among women in Bangladesh must be taken into consideration so that public health interventions may be adopted through appropriate policy.

## INTRODUCTION

Due to economic situation of developed countries, health concern mainly on overweight receives graver attention. The ‘dual burden’ of health concern caused through both underweight and overweight is significantly important for public health policy in developing nations to address (1–3). Studies confirm that these days the problem of overweight in developing nations is gradually taking the driving seat superseding the concerns of underweight due to changes of food habit and lifestyle and working culture (1). There is evidence that this increase has been faster in the developing countries (2). The positive relationship between obesity and socioeconomic position in developing countries stood in sharp contrast with the inverse association observed in developed countries where the prevalence of obesity is higher among women from low socioeconomic groups (3).

A landmark review of studies on socioeconomic status and obesity supports the view that obesity in the developing world would be essentially a non-communicable disease of the socioeconomic elite (2). The problem relating to body mass index (BMI) for both men and women should receive equal attention. However, concerns relating to women in developing countries deserve extra attention because of cultural and economic backdrops, which hinder the blanching between male and female counterparts. An augmented number of literature asserts that an increased BMI of women is independently associated with increasing risk of adverse obstetric and neonatal outcome (4–6). The risks of overweight also include diabetes mellitus, increased risk of cardiovascular disease, cancer, hypertension, and other medical problems (7–16). Besides, a low BMI is often associated with low nutritional status and adverse health outcomes (12), such as preterm birth (17,18), low birthweight (17), mental health impairment (19), increased risk of early mortality (20), and higher risk of infant mortality (14). Early and late stillbirths are also associated with underweight mothers compared to their normal-weight counterparts. Anaemia is also associated with maternal underweight (9). Low pre-pregnancy BMI and short stature of women are known risk factors of poor maternal and birth outcomes. In developing countries, like Bangladesh, maternal underweight is a leading risk factor of preventable death and diseases.

Both lean and obese women carry a risk of adverse pregnancy outcome and overall poor maternal and child health status. Thus, there is growing recognition of a ‘double burden’ of malnutrition among populations in both affluent and less-affluent countries (21), i.e. the co-existence of undernutrition (e.g. stunting or underweight) with overweight, which has been observed at the national and household levels (22); this suggests the necessity of population-based assessments of the patterns, prevalence, and determinants of underweight and overweight among women of reproductive age. Using BMI as an important indicator of nutritional status, it is our aim to examine the nature of the relation between individual sociodemographic category and nutritional status among married women of Bangladesh and also investigate to what extent the factors influence the women to be underweight and overweight.

## MATERIALS AND METHODS

Data for this study have been used from the most recent 2011 Bangladesh Demographic and Health Survey (BDHS) (23). A nationally-representative household-based sample was created through a stratified and two-stage cluster-sampling strategy. A uniform sampling design was adopted across all regions with urban and rural samples drawn separately and in proportion to the population of the regions, unless oversampling was required for any region or group. For both urban and rural areas, geographic sampling units were obtained, and random sampling of households was done in chosen units. The survey provides consistent and reliable estimates of fertility, age at first marriage, family planning, utilization of maternal and child healthcare services, nutrition of children and adults, maternal and child health, knowledge and awareness about sexually transmitted diseases (STDs)/sexually transmitted infections (STIs), HIV/AIDS, and other health-related indicators at the national as well as the regional levels.

The 2011 BDHS gathered information on 17,749 ever-married women aged 15-49 years. The women with various missing information and those who were pregnant during the survey were excluded from analysis. After excluding women with missing information (n=407) on the outcome measure and those who were pregnant (n=1,069) during the survey, the final sample for analysis stood at 16,273. Table 1 provides descriptive characteristics of the weighted sample that have been tabulated according to nutritional status for variables considered in the study.

### Outcome measure

Women's weight status, indicated by their BMI category, was used as the outcome variable in the analyses. BMI was calculated as weight in kg divided by height in metre squared. This measurement of BMI is generally considered an appropriate method for epidemiological studies where objective measurement is less feasible. The well-trained field staff during at-home interviews measured body-size. Weight was measured using an electronic scale with a precision of 0.1 kg, and height was measured with an adjustable measuring-board designed for use in survey settings, which can provide accurate measurements to the nearest 0.1 cm. The BMI was presented in the raw dataset of the survey. The WHO consultants suggest seven categories of BMI cutoff points for Asian countries (24): <16.00 kg/m^2^ (severe underweight), 16·00-16·99 kg/m^2^ (moderate underweight), 17·00-18·49 kg/m^2^ (mild underweight), 18·50-22·99 kg/m^2^ (normal weight), 23.00-24.99 kg/m^2^ (pre-overweight), 25.00-29·99 kg/m^2^ (overweight), and ≥30.00 kg/m^2^ (obese). Given the identification of a BMI of 23.00 as a public-health cutoff for risk of obesity in Asian populations (24) and the emerging evidence suggesting that lower cutoffs are appropriate for populations from the Indian Subcontinent (2,25,26), we narrowed the normal BMI range of 18.50-24.99 to 18.50-22.99. Following the abovementioned literature on Asian countries, we used three grades of underweight and overweight.

**Table 1.** Definition of variables and percentage distribution of ever-married women*(N=16,273) aged 15-49 years, BDHS 2011

Exposure variable	Description	Measurement scale	No.	%
Demographics				
		Ordinal		
Current age (completed years)	Current age of the women at the time of survey	1=15-24	4,674	28.7
		2=25-34	5,564	34.2
		3=35-49	6,035	37.1
		Ordinal		
Current marital status	Respondent's marital status at survey time	1=Married	15,199	93.4
		2=Widowed/Divorced/Separated	1,074	6.6
		Ordinal		
Age at first childbirth (years)	Respondent's age at first livebirth of child	1=<18	8,050	53.7
		2=18+	6,928	46.3
		Ordinal		
Parity	Number of children ever born	1=<3	8,702	53.5
		2=3+	7,571	46.5
		Ordinal		
No. of children aged ≤5 years	Total number of children aged 5 years or below at the household	1=None	7,950	48.9
		2=One	6,018	37.0
		3=Two+	2,305	14.2
Socioeconomics				
		Ordinal		
Women's education	Women's level of education	0=No education	4,653	28.6
		1=Primary	4,889	30.0
		2=Secondary	5,581	34.3
		3=Higher	1,151	7.1
		Binary		
Employment status	Whether respondent was employed at the time of survey	0=Not employed	14,096	86.6
		1=Employed	2,177	13.4
		Ordinal		
Wealth index	Availability of luxurious materials in the household	1=Poorest	2,976	18.3
		2=Poorer	3,169	19.5
		3=Middle	3,265	20.1
		4=Richer	3,390	20.8
		5=Richest	3,473	21.3
		Ordinal		
Food security	Whether the household had deficiency of food	1=Secure	10,899	67.0
		2=Mild insecurity	3,621	22.3
		3=Moderate insecurity	1,269	7.8
		4=Severe insecurity	480	3.0
Environmental				
		Ordinal		
Place of residence	Current place of residence	1=Urban	4,248	26.1
		2=Rural	12,025	73.9
		Ordinal		
Region	Place of region	1=Barisal	886	5.4
		2=Chittagong	2,928	18.0
		3=Dhaka	5,236	32.2
		4=Khulna	2,018	12.4
		5=Rajshahi	2,442	15.0
		6=Rangpur	1,908	11.7
		7=Sylhet	854	5.2
Total			16,273	100.0

*Total number of women by different categories of exposure variables may not always be 16,273 due to missing cases

### Exposure variables

The study considers demographic, socioeconomic and environmental factors to assess the nutritional status of the study women. The effect of one variable on the prevalence of malnutrition is likely to be confounded with the effects of other variables. Therefore, demographic, socioeconomic and environmental characteristics were controlled statistically. The variables included as covariates are: women's current age at interview, current marital status, age at first childbirth, parity, number of children aged ≤5 years in the household, women's education, place of residence, the region, wealth quintile, and food security.

Notably, measurement of poverty and food security is complex and debatable, particularly due to the unavailability of direct and reliable information on household income or expenditure in cross-sectional sample surveys, like BDHS. Despite this, the 2011 BDHS has used wealth index in a conventional way as a proxy measure of socioeconomic status and food security for the first time. The relative index of household wealth was calculated on the basis of a standard set of interviewer-observed assets, including the ownership of consumer items and dwelling characteristics. Besides, food security was measured by the response provided by the respondents to the question, “Did you ask food from relatives or neighbours in the past 12 months directly?” The operational definition and categories of the variables are given in Table 1.

### Statistical analyses

The weighted prevalence of severe underweight, moderate underweight, mild underweight, normal weight, pre-overweight, overweight, and obesity was obtained for each category using the national weights assigned by the cluster design at the primary sampling unit level. The chi-square tests were applied to study the difference in proportions of different BMI between the categories of the exposure variables. The checking of multicollinearity by the estimated variance inflation factor (VIF) shown in Table 2 from multivariable linear regression model reveals its non-existence (27).

To assess the net effect of exposure variables on the outcome measures, multinomial logistic regression analysis was contemplated to be suitable as the outcome measure is polychotomous by nature. The multinomial logistic model is the extension of the binary logistic regression model to outcome measure with *j*=1, 2, 3, …, *k* nominal outcomes. In its general form, the probability of an actor *i* belonging to category *j* is given by the following formula (28):


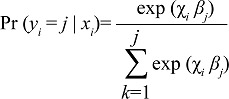


where χ*_i_* is a vector containing the values of *m* covariates for person *i,* and *β_k_* is a vector of *m* +1 parameters (*β*_0_*_k,_ β*_1_*_k,_ β*_2_*_k_ …, β_mk_*) for each *k*=1, 2, 3, …, *j*. To identify the parameters, it is common to choose one reference category and set the corresponding vector of parameters equal to a vector of zeroes. As we did not find substantial differences in the patterning of the sociodemographic and environmental exposures between the different grades of underweight, we merged the different grades of underweight to one category of BMI <18.50 labelling by ‘underweight’. Similarly, we merged overweight and obesity by labelling as ‘overweight’. The results of the multinomial logistic regression analyses have been shown by odds ratios (ORs) with 95% confidence interval (CI) for easy understanding. The level of significance was set at 10%. Data were analyzed using SPSS (version 21) (SPSS Inc., Chicago, IL, USA).

### Ethical issue

The study was based on a nationally-representative anonymous dataset for public use, with no identifiable information on the survey participants. Moreover, the survey followed all protocols prescribed by the WHO and was implemented by National Institute for Population Research and Training (NIPORT) under the Ministry of Health and Family Welfare, Government of Bangladesh. Therefore, no ethics statement is required for this study.

## RESULTS

### Characteristics of the sample

Most of the respondents were rural residents and were currently in union, with mean±SD age of 31.2±9.2 years. The mean age at first marriage, mean age at first childbirth, mean number of children ever born per ever-married woman was 15.6±2.9 years, 17.8±3.3 years, and 2.7±1.9 (not shown in Table 1). Only a few proportions of women had higher education and were employed during survey. The large proportion of the respondents was from Dhaka region. Almost 11% reported to have deficiency of food whereas five categories of wealth quintiles demonstrated nearly equal distribution of women.

**Figure 1.**
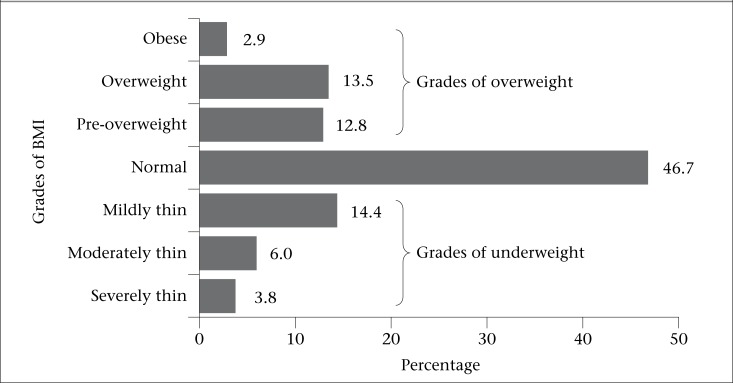
Distribution of ever-married women of reproductive age by different strata of BMI, BDHS 2011

### BMI and sociodemographic factors

The distribution of women across categories of BMI shows that 46.7% were normal, 24.2% were underweight, 12.8% were pre-overweight, and 16.4% were overweight and obese (Figure 1). The associated p values in chi-square analysis suggest significant difference (p<0.01) in seven categories of BMI by different groups of exposure variables, except for the women's employment status (Table 3). The estimated coefficients of the multivariable regression model show significant positive relationship between BMI and current age (p<0.001), women's level of education (p<0.001), and wealth index (p<0.001) whereas negative association was observed for current marital status (p<0.001), age at first childbirth (p<0.001), parity (p<0.001), number of children aged ≤5 years in household (p<0.001), residence (urban to rural) (p<0.001), and food security (secure to insecure) (p<0.001). Region was also a significant factor (p<0.05) for nutritional status of women.

**Table 2.** Multivariable regression coefficients and the variance inflation factor (VIF) for predictors with BMI as outcome measure adjusted for duration of marriage

Predictor	Coefficient	SE	*t*-value	p value	VIF
Age	0.087	0.005	16.635	0.000	2.721
Current marital status	-0.818	0.122	-6.691	0.000	1.097
Age at first childbirth	-0.026	0.010	-2.646	0.008	1.343
Parity	-0.174	0.024	-7.355	0.000	2.179
No. of children aged ≤5 years	-0.212	0.046	-4.651	0.000	1.345
Women's level of education	0.383	0.039	9.774	0.000	1.656
Employment status	-0.052	0.086	-0.602	0.547	1.074
Wealth quintile	0.760	0.027	28.275	0.000	1.808
Food security	-0.140	0.040	-3.485	0.000	1.218
Place of residence	-0.823	0.074	-11.082	0.000	1.326

SE=Standard error

### Trends in BMI by cutoff points

**Figure 2.**
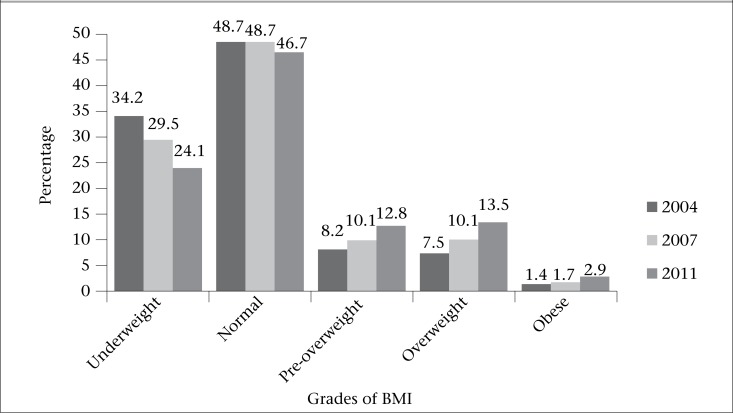
Trends in BMI of ever-married women of reproductive age for the period 2004-2011 by Asia-Pacific standard

Figure 2 shows that the prevalence of normal weight was steady in the period 2004 to 2007 and then decreased by 2.0% from 48.7% to 46.7% during 2007 to 2011. We noted significant decrease of the pervasiveness of underweight for the same period. The prevalence of underweight decreased by 9.9%—from 34.2% to 24.1% during 2004 to 2011. The figure exhibits an apparent increasing trend in all grades of overweight. For instance, the prevalence of pre-overweight increased by 4.2%—from 8.2% to 12.4%—whereas the rate of overweight and obesity increased by two folds during the period 2004-2011. Markedly, the addition of all grades of overweight reveals that the prevalence of overweight exceeded the underweight body-shape of women in 2011.

### Determinants of underweight, pre-overweight, and overweight

Table 4 shows the adjusted ORs for underweight, pre-overweight, and overweight relative to the normal weight for the covariates considered for analysis. Compared to the elder, the younger were more likely to be underweight. Age was positively associated with pre-overweight, overweight, and obesity, relative to normal weight. The likelihood to be underweight was significantly lower for the currently-married women than those who were not in union during the survey. The current marital status was no longer a significant factor for pre-overweight and overweight, controlling for other covariates. The women who had given first childbirth before the age of 18 years were less likely to be underweight, although it showed to have no significant effect on other categories of BMI. The women with fewer children aged ≤5 years at the household were less likely to be underweight and more likely to be overweight than those who had more.

Women's level of education was significantly and negatively associated with being underweight and was positively associated with pre-overweight and overweight relative to normal weight. The wealth quintiles show familiar fashion in the likelihood of being underweight and overweight. The risk of being underweight increased systematically with the decreases in wealth index. In contrast, the risk of being pre-overweight and overweight decreased significantly with the increase of standard of living index. The higher the food security, the lower the risk of being underweight. However, food security had no longer significant effect of being pre-overweight and overweight.

The rural women, compared to their urban counterparts, were more likely to be underweight whereas urban women were more tended to be overweight than their rural counterparts. The women living in the Barisal, Chittagong, Dhaka, Khulna, Rajshahi and Rangpur regions were less likely to be underweight compared to the reference region of Sylhet. Besides, the women from Chittagong, Khulna and Rajshahi regions were more tended to be pre-overweight and overweight than those of the reference region. In addition, compared to the women from Sylhet region, those living in Chittagong and Khulna were significantly at increased risk of overweight.

**Table 3.** Distribution of ever-married women across categories of BMI by different predictors, BDHS 2011

Background characteristics	BMI (kg/m^2^) category							χ^2^
	<16.00	16.00-16.99	17.00-18.49	18.50-22.99	23.00-24.99	25.00-29.99	≥30.00	
Current age (completed years)								590.54^a^
15-24	3.5 (165)	7.9 (371)	19.0 (887)	52.6 (2,457)	9.1 (424)	6.8 (319)	1.1 (51)	
25-34	3.0 (165)	5.4 (298)	12.4 (692)	45.6 (2,536)	14.5 (809)	16.1 (895)	3.0 (169)	
35-49	4.7 (284)	5.0 (300)	12.5 (756)	43.2 (2,609)	14.2 (858)	16.3 (982)	4.1 (247)	
Current marital status								65.75^a^
Married	3.6 (549)	5.8 (877)	14.0 (2,126)	47.2 (7,166)	13.0 (1,976)	13.6 (2,063)	2.9 (442)	
Widowed/Divorced/Separated	6.1 (65)	8.6 (92)	19.5 (209)	40.5 (435)	10.7 (115)	12.4 (133)	2.3 (25)	
Age at first childbirth (years)								112.26^a^
<18	3.9 (316)	6.1 (488)	15.2 (1,225)	48.4 (3,893)	12.4 (999)	11.5 (926)	2.5 (203)	
18+	3.8 (261)	5.5 (384)	12.7 (882)	44.4 (3,073)	13.7 (950)	16.4 (1,134)	3.5 (243)	
Parity								58.78^a^
<3	2.8 (240)	6.2 (541)	14.3 (1,248)	46.9 (4,084)	13.2 (1,151)	13.8 (1,204)	2.7 (234)	
3+	4.9 (373)	5.7 (428)	14.4 (1,087)	46.5 (3,518)	12.4 (939)	13.1 (992)	3.1 (233)	
No. of children aged ≤5 years								181.55^a^
None	3.8 (304)	5.3 (421)	12.8 (1,015)	45.0 (3,575)	14.2 (1,132)	15.5 (1,230)	3.4 (274)	
One	3.8 (228)	6.4 (387)	14.5 (872)	48.3 (2,910)	12.2 (734)	12.3 (741)	2.4 (145)	
Two+	3.5 (82)	7.0 (161)	19.4 (448)	48.5 (1,117)	9.7 (225)	9.8 (225)	2.1 (47)	
Women's education								689.78^a^
No education	5.7 (267)	6.7 (310)	17.2 (801)	48.1 (2,239)	11.2 (521)	9.3 (430)	1.8 (83)	
Primary	4.0 (195)	7.2 (350)	15.5 (756)	48.2 (2,356)	11.7 (572)	11.4 (559)	2.1 (101)	
Secondary	2.6 (143)	5.1 (285)	12.7 (711)	46.0 (2,567)	13.9 (773)	16.0 (890)	3.8 (211)	
Higher	0.8 (9)	2.0 (23)	5.8 (67)	38.2 (440)	19.5 (224)	27.5 (316)	6.2 (71)	
Employment status								6.53
Not employed	3.7 (524)	6.0 (844)	14.5 (2,039)	46.9 (6,607)	12.7 (1,796)	13.3 (1,876)	2.9 (410)	
Employed	4.1 (89)	5.7 (125)	13.6 (297)	45.7 (995)	13.5 (295)	14.7 (320)	2.6 (57)	
Wealth index								2,586.34^a^
Poorest	7.6 (225)	10.2 (304)	22.1 (658)	49.0 (1,458)	6.2 (186)	4.6 (136)	0.3 (9)	
Poorer	4.7 (149)	7.4 (235)	17.9 (568)	53.7 (1,700)	9.7 (308)	6.0 (191)	0.6 (18)	
Middle	3.6 (119)	6.4 (208)	15.4 (503)	50.4 (1,546)	13.1 (427)	9.9 (322)	1.2 (41)	
Richer	2.6 (88)	4.6 (154)	12.3 (417)	45.2 (1,531)	15.2 (514)	17.3 (586)	2.9 (98)	
Richest	0.9 (33)	1.9 (68)	5.5 (189)	36.5 (1,266)	18.9 (655)	27.7 (961)	8.6 (300)	
Food security								498.30^a^
Food-secure	3.1 (334)	4.9 (533)	12.7 (1,382)	45.6 (4,973)	14.2 (1,549)	15.8 (1,727)	3.7 (401)	
Mild insecurity	4.8 (173)	7.4 (266)	16.5 (596)	49.3 (1,784)	10.9 (395)	9.6 (349)	1.5 (56)	
Moderate insecurity	5.9 (74)	10.5 (133)	19.0 (241)	49.5 (628)	7.9 (100)	6.8 (86)	0.6 (4)	
Severe insecurity	6.7 (32)	7.7 (37)	24.2 (116)	44.2 (212)	9.6 (46)	7.2 (35)	0.5 (2)	
Place of residence								940.57^a^
Urban	1.9 (81)	2.9 (123)	8.7 (368)	41.4 (1,759)	16.5 (701)	22.3 (945)	6.4 (271)	
Rural	4.4 (533)	7.0 (846)	16.4 (1,968)	48.6 (5,843)	11.6 (1,390)	10.4 (1,251)	1.6 (196)	
Region								239.28^a^
Barisal	4.5 (40)	6.6 (58)	15.7 (139)	48.9 (433)	11.9 (105)	10.7 (95)	1.8 (16)	
Chittagong	3.3 (96)	4.7 (138)	14.2 (416)	45.5 (1,332)	14.6 (427)	14.5 (426)	3.2 (93)	
Dhaka	4.1 (217)	6.0 (314)	13.4 (704)	45.9 (2,403)	12.5 (653)	14.4 (755)	3.6 (190)	
Khulna	2.2 (45)	4.5 (92)	12.1 (244)	46.7 (943)	14.7 (296)	16.6 (334)	3.2 (64)	
Rajshahi	3.6 (87)	6.7 (164)	14.4 (351)	46.5 (1,135)	13.4 (328)	13.1 (319)	2.4 (59)	
Rangpur	3.6 (69)	6.9 (131)	16.4 (313)	51.9 (990)	10.6 (202)	9.2 (175)	1.4 (27)	
Sylhet	7.0 (60)	8.3 (71)	19.7 (168)	42.7 (365)	9.3 (79)	10.8 (93)	2.1 (18)	
Total	3.8 (614)	6.0 (969)	14.3 (2,336)	46.7 (7,601)	12.8 (2,091)	13.5 (2,196)	2.9 (467)	

Chi-square tests for the cross-tabulation between each variable and the seven categories of BMI

^a^p<0.001

## DISCUSSION

The present study, to our knowledge, examines for the first time the sociodemographic and environmental distribution of nutritional status of ever-married women with mean age of 31.2±9.2 years, using the Asia-specific cutoff points of BMI. The findings document the co-existence of dual burden of underweight and overweight among women in Bangladesh, using data of the most recent nationally-representative population sample survey covering all regions of Bangladesh. Findings show that, in 2011, the prevalence of underweight, normal weight, and overweight was 24.1%, 46.7%, and 29.2% respectively, suggesting that the incidence of overweight exceeded underweight for the first time. The incidence of underweight has decreased substantially and continually during the period from 2004 to 2011.

There is an apparent socioeconomic, demographic and environmental distribution of causal patterns of nutritional status, with younger women, women from low socioeconomic position, and rural residents experiencing a greater risk of underweight, and the elder women, those from high socioeconomic status, and urban residents experiencing the greatest risk of being pre-overweight, overweight, and obese. Our findings are consistent with earlier studies conducted on women and men from the neighbouring country India (2,29–32) and other developing countries (33,34).

The increased likelihood of being underweight and decreased risk of being overweight among the younger women, compared to their elder counterparts, and decreased risk of underweight among women who were currently in union may be partly attributed to physical activity, cultural norms, and food practising. Biological phenomena are also concerned with undernutrition of young women compared to their elders. The ever-married women who were not currently in union during the survey were possibly older than were those who were currently in union. The martially-disrupted women in Bangladesh may have less physical activity than those who live with husbands because of limited outdoor activities due to specific climatic and social circumstances. Our findings are supported by earlier studies (32,33). Studies pointed that, although individual behaviours, such as physical activity and sound nutritional practices, have been demonstrated to lead to weight loss, scaling up of these messages to the population level has proven elusive (35). Increasingly, those responsible for disease prevention have come to emphasize that individual-level behaviours are not sufficient, by themselves, to mitigate the worldwide obesity epidemic (36). When the parity and total number of children aged ≤5 years in household are concerned, the latter one appeared as a more powerful predictor of women's nutritional status than the previous one. For instance, women with low parity (<3) were significantly more tended to be pre-overweight and overweight, relative to normal whereas the women with fewer children were less likely to be underweight and more tended to be obese, including pre-overweight and overweight. In patriarchal society, like Bangladesh, where childrearing is the main responsibility of women, the adverse lifestyle risk factors associated with rearing of many children at a time significantly affect the women's health through a complex way, which includes socioeconomic, demographic, mental, psychological and also biological factors. However, consistent with earlier studies (37), our findings show significant negative association between parity and number of children aged five or below in the household and BMI.

**Table 4.** Adjusted odds ratios (95% CI) for the study variables and covariates from multinomial logistic model predicting underweight, pre-overweight, overweight, and obesity for the ever-married women

Background characteristics	BMI category		
	Underweight vs Normal weight	Pre-overweight vs Normal weight	Overweight/Obese vs Normal weight
Current age (completed years)				
15-24	1.30 (1.12-1.51)^a^	0.47 (0.39-0.58)^a^	0.29 (0.24-0.36)^a^
25-34	0.95 (0.85-1.06)	0.91 (0.80-1.04)	0.81 (0.71-0.91)^b^
35-49	Reference	Reference	Reference
Current marital status			
Currently married	0.64 (0.54-0.75)^a^	1.09 (0.86-1.37)	1.01 (0.82-1.25)
Widowed/Divorced/Separated	Reference	Reference	Reference
Age at first childbirth (years)			
<18	0.93 (0.85-1.02)^d^	1.06 (0.95-1.18)	0.96 (0.87-1.07)
18+	Reference	Reference	Reference
Parity			
<3	0.95 (0.85-1.06)	1.24 (1.09-1.41)^a^	1.10 (0.97-1.24)^d^
3+	Reference	Reference	Reference
No. of children aged ≤5 years			
None	0.81 (0.71-0.92)^a^	1.34 (1.12-1.69)^a^	1.34 (1.13-1.59)^a^
One	0.87 (0.77-0.98)^b^	1.21 (1.02-1.44)^c^	1.19 (1.00-1.40)^c^
Two+	Reference	Reference	Reference
Women's education			
No education	1.78 (1.32-2.40)^a^	0.79 (0.62-1.01)^c^	0.59 (0.48-0.74)^a^
Primary	1.73 (1.29-2.31)^a^	0.79 (0.63-1.00)^c^	0.69 (0.56-0.85)^a^
Secondary	1.52 (1.14-2.02)^b^	0.86 (0.69-1.06)	0.86 (0.71-1.03)^d^
Higher	Reference	Reference	Reference
Wealth index			
Poorest	2.75 (2.27-3.35)^a^	0.29 (0.23-0.36)^a^	0.15 (0.12-0.19)^a^
Poorer	2.00 (1.65-2.42)^a^	0.41 (0.33-0.49)^a^	0.17 (0.14-0.20)^a^
Middle	1.88 (1.56-2.26)^a^	0.54 (0.45-0.64)^a^	0.28 (0.24-0.33)^a^
Richer	1.68 (1.41-2.02)^a^	0.69 (0.59-0.81)^a^	0.53 (0.46-0.61)^a^
Richest	Reference	Reference	Reference
Food security			
Food-secure	0.75 (0.60-0.93)^b^	0.96 (0.68-1.36)	1.14 (0.78-1.67)
Mild insecurity	0.79 (0.63-0.99)^c^	0.89 (0.63-1.27)	1.03 (0.70-1.51)
Moderate insecurity	0.84 (0.66-1.07)	0.74 (0.50-1.10)	0.86 (0.56-1.33)
Severe insecurity	Reference	Reference	Reference
Place of residence			
Urban	0.80 (0.71-0.91)^a^	1.09 (0.96-1.25)	1.33 (1.18-1.51)^a^
Rural	Reference	Reference	Reference
Region			
Barisal	0.70 (0.55-0.89)^a^	1.23 (0.88-1.73)	1.12 (0.80-1.55)
Chittagong	0.62 (0.51-0.76)^a^	1.44 (1.09-1.90)^b^	1.35 (1.04-1.75)^b^
Dhaka	0.71 (0.59-0.85)^a^	1.05 (0.80-1.38)	1.05 (0.82-1.36)
Khulna	0.52 (0.42-0.65)^a^	1.35 (1.01-1.81)^c^	1.52 (1.16-1.99)^a^
Rajshahi	0.67 (0.55-0.81)^a^	1.33 (0.99-1.77)^c^	1.37 (1.05-1.80)^a^
Rangpur	0.60 (0.49-0.74)^a^	1.08 (0.80-1.46)	0.97 (0.72-1.29)
Sylhet	Reference	Reference	Reference

^a^p<0.001

^b^p<0.01

^c^p<0.05

^d^p<0.10

Findings reveal a strong significant association between BMI and women's level of education and wealth quintile. The higher the level of education and the wealth quintile, the lower was the risk of being underweight. In contrast, the grades of overweight increased significantly with increase in women's level of education and wealth quintile. The increased risk of grades of overweight for women in more flattering social position is partially attributed to the capability of boasting up more-than-adequate food supplies and lesser physical activities than women belonging to poor socioeconomic status. The higher incidence of overweight and obesity in higher socioeconomic groups may be partly explained by the possible indifference to body-shapes. These differences may contribute to explaining the strong observed association between high socioeconomic positions and overweight (2).

Moreover, these days people with increased income are willing to pay for a larger number of high-calorie beverages to respond to the elite marketing, which substantially contribute to gain weight. Thus, in spite of socioeconomic variability in population, the increased consumption of high-calorie beverages may explain some of the positive relationship between wealth quintile and BMI (38). Consistent with many studies (2,29–34), ours suggest that women belonging to higher socioeconomic position are at the elevated risk of being overweight, and those with poor socioeconomic status are at increased risk of underweight, further suggesting the ‘dual burden’ of nutritional status of women in Bangladesh. A similar result was found in an earlier study in analyzing socioeconomic and geographic patterning of under- and overnutrition among women in Bangladesh (39).

The unavailability of food is strongly associated with underweight body-shape of women and not significantly with overweight. Whereas obesity is associated with privileged circumstances in low-income countries (40), it is more often associated with lower socioeconomic status in developed countries (41). This observation suggests that, in a more food-abundant environment, socioeconomic factors, such as wealth, education, and income level, may change the relationship between food availability and overweight and/or obesity. In the low- and middle-income countries, these relationships are less consistent (40). Obesity may be associated with wealth in some situations but with poverty in others (42). Underweight and overweight may, sometimes, co-exist in the same neighbourhoods (22), or even in the same households (43). However, our findings are quite in a good agreement with that of low-income countries (40).

The association between socioeconomic status and BMI in low-income settings, like Bangladesh suggests that rural people is likely to be subjected to the changing patterns of food availability, food composition, and consumption behaviour. Findings reveal that rural women were less likely to be overweight than those in urban areas. This finding is similar to that of an Indian study (2). Moreover, we marked significant regional variation of BMI among women. The women living in Sylhet region were at increased risk of underweight relative to normal weight. The previous BDHSs conducted in 2004 and 2007 also reported higher prevalence of underweight among women living in Sylhet region. Further, the women from Chittagong, Khulna, and Rajshahi were at increased risk of overweight and/or obesity than were those from Sylhet region. These findings underscore that women living in the more urbanized regions may be at higher risk of overweight and those living in the typically agriculture-based region may be at risk of being underweight. Although the region of Dhaka is more exposed to urbaniation, the rapid migration of rural people to Dhaka, the capital city of Bangladesh, may have attenuated its effect on being overweight or obese among women. This finding is also consistent with an earlier study on Bangladeshi women (39).

### Strengths and limitations

The study has several strengths and limitations that urge explanations. The first limitation is that, we used BMI as the only measure of overweight. Although BMI is a reliable indicator of body fatness and a low BMI is likely to be a valid proxy for chronic energy deficiency, BMI does not distinguish between body fat and lean body mass. Waist-circumference (26) and waist-to-hip ratios (44) consequently have been suggested as better markers of obesity. Recent studies have shown that South Asians have the poorest correlation between waist-circumference and BMI when comparing them against the Europeans, Chinese, and Aboriginal persons, although the correlation is still substantial (25). However, BMI is a WHO-prescribed indicator of nutritional status measurement that is internationally used. Second, the BMI cutoff points we used in this study may understate health risk. It is notable that, a growing body of literature, including those on Indians (2,31,32), have used this BMI cutoff points, which is recommended by WHO and now globally recognized. Third, as a cross-sectional study, the present analysis is limited to its ability to elucidate causal relationships between risk factors and overweight. BMI can overestimate body fat in individuals who are very muscular and underestimate body fat in individuals who have lost muscle mass, such as many elderly (45). Despite these, the strengths of the study include the large sample consisting of both rural and urban populations, representativeness of the national population, and information on potential determinants of underweight and overweight.

### Conclusions

Bangladesh is facing the dual burden of underweight and overweight. The findings suggest that the nutritional status of women is related to individual, socioeconomic and environmental level. The nutritional status of individuals and societies, at a given point in time, is likely to reflect the cumulative synergy between physiologic endowments and the social environment (2). It is likely that the identification of sociodemographic and environmental factors affecting nutritional status of women is of great importance for developing and targeting interventions to face the dual burden of underweight and overweight as these epidemics may adversely impact the health of both mother and child in the long run and thereby the society as a whole. Regardless, systematic and regular monitoring and surveillance of the social trajectory of nutritional status of women and men in Bangladesh is crucial to develop apposite strategy that addresses the persistent and chronic problem of underweight and the emerging problem of overweight. The dual existence of both types of malnutrition among women in Bangladesh must be taken into consideration so that public health interventions may be adopted through appropriate policy.

**Conflict of interest:** Authors declare no conflicts of interest.
